# Identification of Tea Plant Purple Acid Phosphatase Genes and Their Expression Responses to Excess Iron

**DOI:** 10.3390/ijms20081954

**Published:** 2019-04-21

**Authors:** Chaoyan Yin, Fei Wang, Huiqin Fan, Yanming Fang, Wenfeng Li

**Affiliations:** Co-Innovation Center for Sustainable Forestry in Southern China, College of Biology and the Environment, Nanjing Forestry University, Nanjing 210037, China; yinchaoyan@gmail.com (C.Y.); wangfei3150035@163.com (F.W.); f802904@163.com (H.F.); jwu4@njfu.edu.cn (Y.F.)

**Keywords:** PAP, conserved motif, transcriptome, Fe, *Camellia sinensis*

## Abstract

Purple acid phosphatase (PAP) encoding genes are a multigene family. PAPs require iron (Fe) to exert their functions that are involved in diverse biological roles including Fe homeostasis. However, the possible roles of PAPs in response to excess Fe remain unknown. In this study, we attempted to understand the regulation of PAPs by excess Fe in tea plant (*Camellia sinensis*). A genome-wide investigation of PAP encoding genes identified 19 *CsPAP* members based on the conserved motifs. The phylogenetic analysis showed that PAPs could be clustered into four groups, of which group II contained two specific cysteine-containing motifs “GGECGV” and “YERTC”. To explore the expression patterns of *CsPAP* genes in response to excessive Fe supply, RNA-sequencing (RNA-seq) analyses were performed to compare their transcript abundances between tea plants that are grown under normal and high iron conditions, respectively. 17 members were shown to be transcribed in both roots and leaves. When supplied with a high amount of iron, the expression levels of four genes were significantly changed. Of which, *CsPAP15a*, *CsPAP23* and *CsPAP27c* were shown as downregulated, while the highly expressed *CsPAP10a* was upregulated. Moreover, *CsPAP23* was found to be alternatively spliced, suggesting its post-transcriptional regulation. The present work implicates that some *CsPAP* genes could be associated with the responses of tea plants to the iron regime, which may offer a new direction towards a further understanding of iron homeostasis and provide the potential approaches for crop improvement in terms of iron biofortification.

## 1. Introduction

Purple acid phosphatases (PAPs) are metallohydrolases that use a binuclear metal ion center to catalyze the hydrolysis of a broad range of phosphorylated substrates from acidic to neutral pH [1]. All characterized active PAPs have a heterovalent active site (Fe(III)-M(II), where M = Fe, Zn, or Mn) for catalysis. The charge transitions between a tyrosine amino acid and ferric ion (Fe(III) confer the purple color to the PAPs [2]. PAPs from different species have conserved structural and sequence motifs. The metal-coordinating amino acid residues of PAPs consist of seven invariant residues located in five blocks of conserved amino acid (G**D**XG–G**D**XX**Y**–G**N**H(D/E) –VXX**H**–G**H**X**H**; bold letters indicate metal binding residues). The genome-wide PAP encoding genes have been identified in model plant *Arabidopsis* (*Arabidopsis thaliana*) and important crops including rice (*Oryza sativa*), soybean (*Glycine max*), maize (*Zea mays*), chickpea (*Cicer arietnum*) and physic nut (*Jatropha curcas*) based on the presence of conserved sequence motifs [3,4,5,6,7,8]. While mammalian organisms contain one or two PAPs, there are relatively large numbers of PAP family members in plants [2]. Studies have shown that *Arabidopsis* contains 29 isoforms of this enzyme. The number of putative PAP isozymes has been predicted to be 26 in rice, 35 in soybean, 33 in maize and 25 in chickpea [3,4,5,6].

According to the protein size, PAPs have been categorized as at least two forms: Low molecular mass (LMM, ~35 kDa) and high molecular mass (HMM, ~55–60 kDa) PAPs. The monomeric LMM proteins are carrying only the catalytic domain. The HMM PAPs are homodimeric or heterodimeric proteins, where each subunit has an unknown function N-terminal domain and a C-terminal domain which contains the active site [2]. Previous studies have clustered plant PAPs into three major groups (groups I, II, and III), which consist of eight subgroups (Ia-1, Ia-2, Ib-1, Ib-2, IIa, IIb, IIIa, and IIIb), based on predicted amino acid sequences. Most of the proteins in group I and II contain more than 400 amino acid residues, and all proteins in group III comprise less than 400 residues [5,9].

PAPs are bifunctional enzymes that catalyze both hydrolytic reactions and peroxidations. In recent years, a variety of biological roles of PAPs have been discussed. Mammalian PAPs have been associated with iron transport [10] and the generation of reactive oxygen species (ROS) [11]. Plant PAPs have been proposed to play diverse roles in growth, development and the adaptation to environmental stresses. Overexpression of *AtPAP2* leads to faster growth by modulating carbon metabolism in *Arabidopsis* and *Camelina sativa* [12]. *NtPAP12* was suggested to be involved in the cell wall synthesis by dephosphorylation of wall proteins in tobacco [13]. *PAP24* was identified to be involved in root skewing [14]. *GmPAP3* could improve the tolerance to salt and osmotic stresses by reducing oxidative damage [15]. *PAP5* was found to confer defense responses to the bacterial pathogen *Pseudomonas syringae* [16]. Plant PAPs have been known to play important roles in mineral nutrition homeostasis including phosphorus (P) and iron (Fe). In rice, *OsPAP21b* improved phosphate acquisition and utilization and *OsPAP26* promoted remobilization of phosphate from the senescing leaves [17,18,19]. Overexpressing *AtPAP15* enhanced phosphorus efficiency in soybean and increased yield when grown on acidic soils [20]. *AtPAP7* was robustly regulated by FIT (FER-like Iron Deficiency-Induced Transcription Factor), the central regulator of iron uptake, in *Arabidopsis* [21]. It was found that the iron content was significantly increased in *AtPAP23* overexpression *Arabidopsis* plants [22]. Overexpression of *AtPAP15* led to increased foliar ascorbate and decreased foliar phytate [23]. PAP members from maize, wheat, barley, rice and *Arabidopsis* exhibited specific enzymatic activity against phytate [24,25,26]. Phytate is a salt form of phytic acid (InsP6) that cannot be digested by humans and monogastric animals, and furthermore, it reduced the availability of iron and zinc by forming strong complexes with the metals [27,28]. Therefore, decreasing the phytate content of the edible parts by PAPs will be benefit to human health.

Tea plant (*Camellia sinensis*) is one of the world’s most important beverage crops. Tea plant is mainly cultivated on acidic soils of the tropics and sub-tropics region, where bioavailable phosphate deficiency and iron excess commonly occur due to the biochemical characteristics of elements [29]. Iron is an essential micronutrient for plants and humans. Plants tightly regulate iron uptake to maintain a balance between the demand for growth and the risk of overaccumulation that could lead to cellular damage [30].

Despite few studies that have implied that PAPs might play an important role in iron homeostasis, little is known about the possible functions of tea plant PAPs under excess iron conditions. The aim of the study was to understand the regulation of *PAP* genes in tea plant by Fe status. We first identified PAP encoding genes in tea plant genome and then investigated their expression patterns in response to high iron. We found 19 putative CsPAPs and the phylogenetic analysis indicated that they are clustered into four groups. Further bioinformatic analyses revealed that two conserved motifs “GGECGV” and “YERTC” are uniquely present in the group II PAPs. We performed transcriptome analyses to elucidate genes involved in excessive Fe responses. Our study has shown that extensive genes associating with the oxidation-reduction process and photosynthesis were affected by a high Fe supply. The expression levels of four *CsPAP* genes were significantly changed under high Fe conditions, implying that they might participate in iron homeostasis. Taken together, these results illustrate that the structure variation and expression regulation of *PAP*s are more complex than previously described and provide new information for a comprehensive understanding of plant *PAP*s in the future.

## 2. Results

### 2.1. The Tea Plant Genome Encodes 19 Putative Purple Acid Phosphatases (PAPs)

The keyword “purple acid phosphatase” search hit 21 genes. Further searches using the protein sequences of the 29 *Arabidopsis* PAPs as the queries with BLASTP at the tea plant genome database website resulted in the retrieval of 53 predicted PAPs based on the similarity of protein sequences with E value ≤ e^−10^. 12 duplicates were further removed, which resulted in 62 potential PAPs in total. Among them, 16 PAPs possess the complete set of conserved seven metal-binding residues (D, D, Y, N, H, H, H) of PAP metallosterase in five consensus blocks (Table 1). Another three of the potential tea plant PAPs (CsPAP2, CsPAP29b and CsPAP3b) lack the conserved residue (D) in the first block. In addition, in proteins of CsPAP29a, CsPAP29b and CsPAP29c, one conserved residue in the second block was changed from “Y” to “F”, which also occurred in PAPs from *Arabidopsis*, maize, chickpea and soybean [4,5,6,7]. Furthermore, five out of the 62-candidate tea plant PAPs lack three of the five blocks. Even though their overall amino acid sequences exhibited significant levels of homology to plant known PAPs when BLASTing in NCBI, however, they were considered as inactive PAPs because of the significant lack of active sites and incomplete metallophos domain that conserved in purple acid phosphatases of the metallophosphatase superfamily, which were identified by the Phoibus program (https://www.ebi.ac.uk/Tools/hmmer) (Table 2).

The remaining genes encode products that contain none of the five consensus blocks. Taken together, 19 PAPs with four or five conserved active motifs were identified (Table S1). Next, each of the 19 predicted protein sequences was used as a query sequence for the second round BLASTP search at the same database. However, no more predicted PAPs were found, suggesting that the total number of predicted active *PAP* genes in the tea plant genome was likely to be 19 (Table 1). General information of the deduced CsPAP proteins including predicted size, signal peptide, subcellular location and potential N-linked glycosylation sites were summarized (Table 2).

### 2.2. Phylogenetic Investigations of Tea Plant PAPs

**Figure 1:**
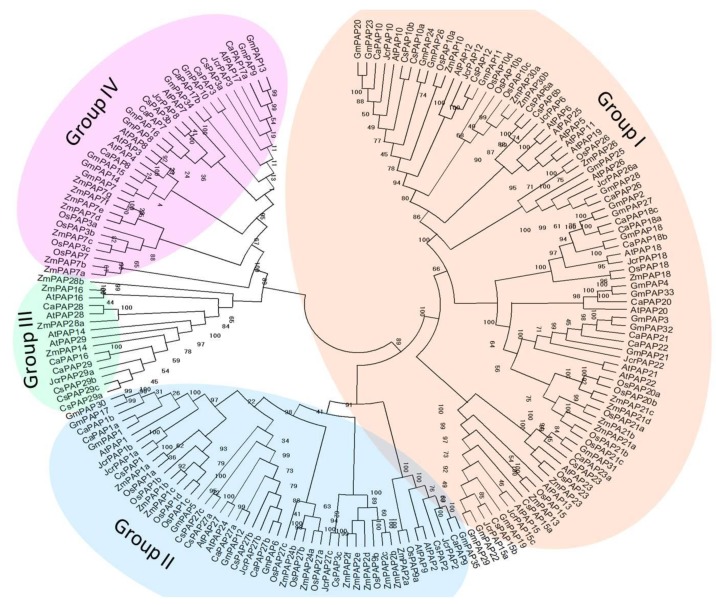
Phylogenetic analysis of purple acid phosphatases (PAPs) from different plant species. Phylogenetic tree of 19 CsPAPs in tea plant, 29 AtPAPs in *Arabidopsis*, 26 OsPAPs in rice, 35 GmPAPs in soybean, 33 ZmPAPs in maize, 18 JcrPAPs in physic nut and 25CaPAPs in chickpea was constructed based on the clustering analysis of their amino acid sequences. The main groups (groups I, II, III and IV) are illustrated.

A neighbor-joining phylogenetic tree was constructed using amino acid sequences of PAPs from *Arabidopsis*, rice, soybean, maize, physic nut and chickpea through a multiple sequence alignment. All of the plant PAPs could be classified into four major groups (groups I, II, III and IV). A further division of the four major groups yielded seven subgroups (Figure 1). Among the four groups, group I contained the most PAPs, and this group can be subdivided into two subgroups I-1 and I-2. Previously identified *Arabidopsis* PAP family Ia and Ib-2 [5] were clustered into subgroups I-1, and Ib-1 was clustered into subgroups I-2. Group II were further divided into two subgroups II-1 and II-2, which included the *Arabidopsis* PAP family IIa and IIb, respectively. Group III was the smallest group and contained *Arabidopsis* PAP members of IIIa; while members of IIIb were classified into group IV. Group IV also consisted of subgroups, PAPs from *Arabidopsis*, soybean, chickpea and tea plant were all presented in the same subgroup, whereas PAPs from monocots, i.e., maize and rice, formed the other subgroups (Figure 1). To investigate the phylogenetic relations of PAPs in monocots and dicots, the phylogenetic tree was constructed using amino acid sequences of all PAP7 and PAP3 homologs from *Arabidopsis*, rice, soybean, maize and chickpea. Except for GmPAP3, which belongs to groups I, all of the 18 PAP7 and PAP3 homologs belong to groups IV and could be classified into three major clades. PAPs from dicots were clustered into the same clade (Figure S1). Members in subgroup IV-2 are LMW PAPs.

### 2.3. Group II PAPs Shared Two Cysteine-containing Conserved Blocks

**Figure 2:**
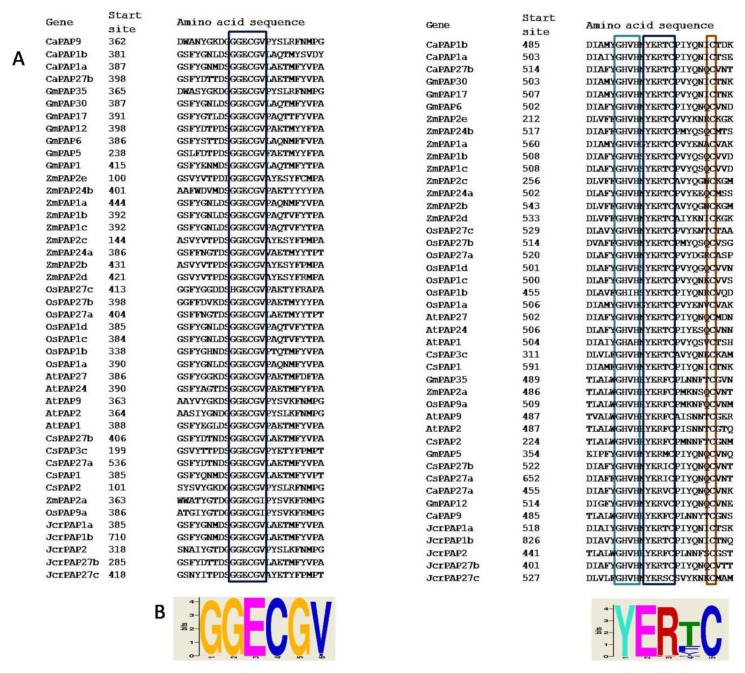
Group II PAPs contain two cysteine-containing conserved motifs. (**A**) Amino-acid alignment of the cysteine-containing conserved motifs in group II PAP members from tea plant (CsPAP), *Arabidopsis thaliana* (AtPAP), soybean (GmPAP), rice (OsPAP), maize (ZmPAP), physic nut (JcrPAPs) and chickpea (CaPAP), conserved residues are boxed; (**B**) logo of the motifs inferred from the sequences of group II PAP members using the MEME suite.

The sequence alignment revealed that two blocks contained invariant residues “GGECGV” and “YERTC” in six CsPAPs which all belong to group II (Figure 2). We then searched the occurrence of these two motifs in PAP families from tea plant, *Arabidopsis*, rice, soybean, maize and chickpea. The result revealed that both conserved blocks were exclusively present in group II PAP members (Figure 2). Each PAP in group II has one “GGECGV” motif except CsPAP1a, which contains two motifs. This motif is located around position 370 in most of the PAPs. This motif is also possessed in some PAPs near position 120. The “YERTC” was located at position 480, which was adjacent to the conserved metal binding motif “GHVH”. In addition, there was a conserved cysteine that is present six residues downstream of “YERTC”.

### 2.4. Transcriptome Analysis of Tea Plant

To gain the expression patterns of *CsPAP* genes and their transcriptional responses to excess iron, we conducted a comparative analysis of transcriptomes of roots and leaves via the RNA-seq technique. Read processing and mapping statistics were shown in Table S2. Based on read mapping results, the transcript abundance was estimated and normalized. Genes with an adjusted *p*-value < 0.05 and fold change > 2 were considered as differentially expressed genes (DEGs). The result showed that 18.5% (6837 out of 36,952) of annotated genes were responsive to excess iron in leaves (Supplementary Data set).

**Figure 3:**
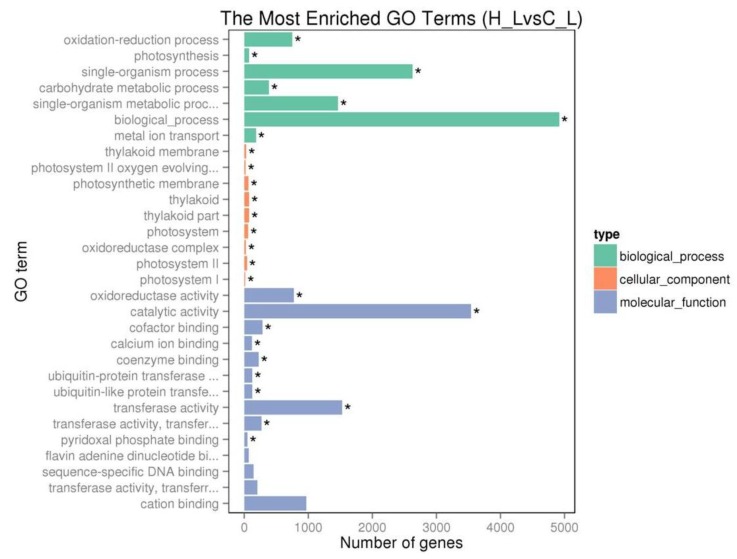
Gene ontology (GO) enrichment analyses of iron excess regulated differentially expressed genes (DEGs). * GO terms that were significantly enriched in the DEGs were summarized in three main categories: Biological process (green), molecular function (blue), and cellular component (orange). The *x*-axis indicates gene numbers in each term.

To understand the relevance of DEGs to different physiological functions based on gene ontology (GO) classification, DEGs were classified according to molecular function, biological process, and cellular component. The GO enrichment analysis revealed that extensive genes associated with important biological processes including the oxidation-reduction process (715 genes), photosynthesis (78 genes) and carbohydrate metabolic process (385 genes) were significantly regulated by excess Fe. The majority of DEGs have molecular functions of the oxidoreductase activity (776 genes), transferase activity (1530 genes), cation binding (972 genes) and catalytic activities (3542 genes) (Figure 3). Furthermore, a set of 385 genes that were involved in oxidation-reduction processes, such as peroxidase and cytochrome P450 encoding genes, were upregulated (Figure S2, Supplementary Data set), suggesting an extensive adjustment for cell redox homeostasis. In addition, 90 iron ion binding genes including ferritins and flavonoid 3’-hydroxylase were increased, implying a strong control for iron homeostasis that was interrupted by high iron (Figure S2, Supplementary Data set). Interestingly, 33 genes functioning in a protein ubiquitination process were upregulated by excess iron. Moreover, 141 transcription factors were induced, including WRKY, GATA and ethylene-responsive transcription factors (Figure S2, Supplementary Data set). Many genes that were involved in photosynthesis and carbohydrate metabolic process, for example genes located in photosystem I, II and thylakoid, were downregulated (Figure S3, Supplementary Data set), indicating a carefully controlled change in photosynthesis under high Fe conditions.

**Figure 4:**
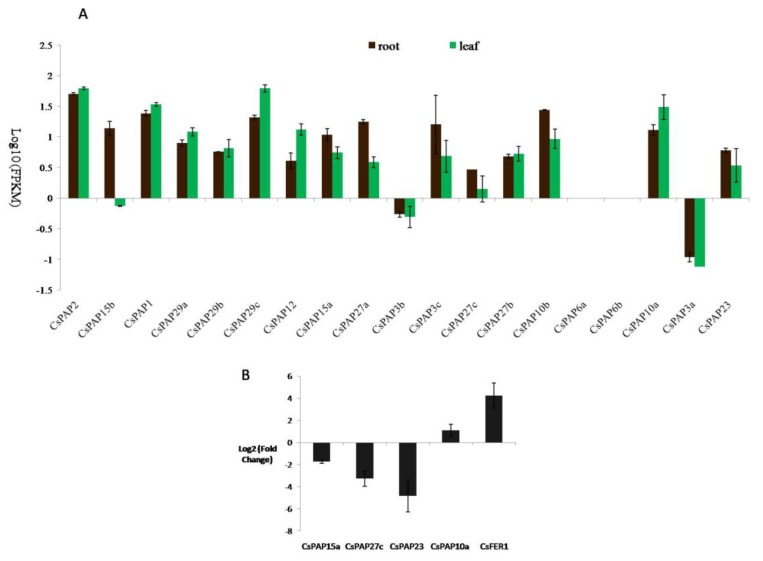
The expression of *CsPAP* genes and their responses to excess iron. (**A**) Expression profiles of 19 *CsPAP*s in roots and leaves in normal conditions by RNA-seq analysis; (**B**) significantly changed *CsPAP*s in leaves by excess iron compared to the normal conditions (*p* ≤ 0.05; fold change > 2). Data in bar charts are the means ± SE of three biological replicates.

Ferritin is an iron-carrying protein in animals and plants. cDNA of *CsFER1* was cloned from tea plant and proved to be a homolog of *AtFer1*, the main ferritin gene in *Arabidopsis* leaves, by DNA sequencing (Table S3). The transcript abundance of *CsFER1* increased by excess iron supply (Figure S4). Therefore, it can be used as a marker gene to indicate the gene expression under excess iron conditions. Similarly, the transcriptomic data results showed that the transcript level of *CsFER1* dramatically increased upon higher iron supply (Figure 4).

### 2.5. The Expression of Tea Plant PAP Genes and Their Responses to Iron Supply

The expression patterns of *CsPAP* genes in roots and leaves were investigated and analyzed using transcriptomic data. Of the 19 *PAP* genes, 17 were expressed in roots and leaves (Figure 4). Expression of the remaining two genes, *CsPAP6a* and *CsPAP6b*, was not detectable with RNA-seq under our conditions. *CsPAP2* and *CsPAP29c* showed the highest abundance, with the expression level of greater than 50FPKM, in leaves. *CsPAP1* and *CsPAP10a* had more than 20FPKM, the second highest expressed genes in leaves. In roots, *CsPAP2* had the greatest expression level with 50.9FPKM. *CsPAP1*, *CsPAP29c* and *CsPAP10b* had more than 20FPKM in roots. *CsPAP3a* and *CsPAP3b* showed a low expression level, less than 1FPKM in both roots and leaves. Notably, *CsPAP15b* had 14.3FPKM in roots, but only 0.7FPKM in leaves (Figure 4A).

Iron plays structural and catalytic roles in PAPs, thus we investigated the transcriptional responses of *PAP* genes to different iron supply conditions (0.032 mM and 32 mM Fe-EDTA, respectively) in tea leaves. 19 *PAP* genes were analyzed using the RNA-seq technique. It demonstrated that four of them were responsive to iron excess. The transcription of three *PAP* genes (*CsPAP15a*, *CsPAP23* and *CsPAP27c*) was repressed under higher iron conditions. However, the significant increase of the *CsPAP10a* transcript level by higher iron treatment was revealed (Figure 4B). For the other *CsPAP* genes, their transcript levels were not obviously affected by a high iron treatment.

### 2.6. CsPAP23 Produced Splice Variants

**Figure 5:**
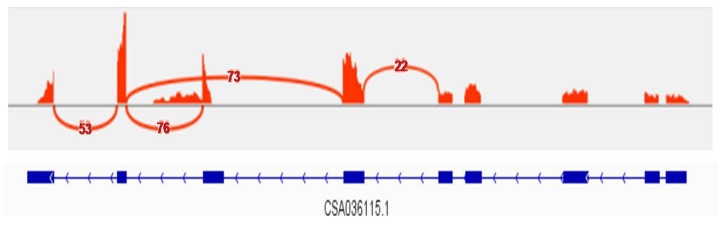
Visualization of the exon-intron structure of alternatively spliced *CsPAP23*. Sashimi plots show the number of RNA-seq reads mapping to loci associated with AS events in roots and leaves. The heights of the bars represent overall read coverage. Genomic coordinates below the plots show the exons (box) and introns (line). Arcs representing splice junctions connect exons. Arcs display the number of reads split across the junction.

The genomic DNA sequence encoding *CsPAP23* consisted of nine exons and eight introns (Figure 5). Reads generated by RNA-seq were mapped onto the reference genome. All nine exons were supported by many reads and most of the introns were not covered by any read. Whereas, it clearly showed that considerable reads mapped onto the 5’ region of intron 7 (Figure 5). This result implied that an intron retention event occurred in *CsPAP23*. In addition, a significant amount of junction reads between exon 6 and exon 8 were detected (Figure 5). This indicated that exon 7 can be skipped. Consequently, the transcription of the *CsPAP23* gene can produce at least three transcripts including constitutive (fully processed) transcripts as well as splice variants in which the intronic sequence was not completely removed, or the exonic sequence was skipped.

## 3. Discussion

Considerable progress has been made in past studies on the structure of PAPs, which allows us to identify PAP encoding genes at genome wide, from both model plant and important crops based on the conserved sequence and structural motifs. Unlike few *PAP*s in mammalian, plants contain multiple genes that form the *PAP* gene family, varying from 15 to 44 across species [31]. We identified 19 *PAP* genes from tea plant, one of the most valuable crops, which is an evergreen shrub. Four different groups with seven subgroups were classified according to the phylogenetic analysis using PAPs encoded by tea plant and those from *Arabidopsis*, rice, soybean, maize, physic nut and chickpea, which represent plant *PAP*s at the genome scale (Figure 2). This clustering scheme was mostly consistent with that of previously established based on *Arabidopsis* PAPs [5]. There was a clear correlation between the clustering of the groups and the size of the PAPs. Group III and IV were composed of five CsPAPs that comprise fewer than 400 amino acids, which was consistent with their structure feature, that is, the lack of N-terminal domain of purple acid phosphatase proteins (Table 2). This finding suggested that these PAPs are likely to have similar biochemical, catalytic and/or functional properties. Group III was expanded in tea plant, notably by the presence of forming a sister clade (Figure 1). For example, these are three genes, *CsPAP29a*, *CsPAP29b* and *CsPAP29c*, that are arranged in tandem repeat on chromosome (Table 2). Exploration of these genes across other germplasm collections would provide further information on their evolution. Group IV contains two subgroups. All members in subgroup IV-1 were from monocots. Homologes of PAP7 and PAP3 from monocots and dicots were likewise clustered into different clades (Figure S1), indicating that PAPs in monocots and dicots might be involved in different roles.

Based on the alignment of amino acid sequences of PAPs, the cysteine-containing conserved blocks “GGECGV” and “YERTC”, as well as a conserved cysteine six residues near the “YERTC” were discovered in group II PAPs. The HMM PAPs are homodimeric or heterodimeric proteins, with each subunit having an unknown function N-terminal domain and a C-terminal domain which contains the active site. The two subunits in the plant PAPs were linked via a disulfide bridge that keeps the structural integrity of the protein and probably increase the stability of the protein. Thus, cysteines probably play an essential role in protein function by maintaining the higher-order structure through the formation of disulfide [32,33]. Indeed, the structure of diphosphonucleotide phosphatase/phosphodiesterase (PPD1) from yellow lupin (*Lupinus luteus*) has revealed that cysteine 484 (C484) in the “YERTC” motif and a cysteine six residues away from it (C491) helped to position the interfacing residues. Cysteine 366 within the “GGECGV” motif in PPD1 was involved in hydrogen bonding to the adjacent subunit [34]. It implicated that the conserved amino acid residues were required for the formation of correct structure in group II PAPs. Notably, the red kidney bean purple acid phosphatase (KBPAP) form a homodimeric glycoprotein with only one disulfide bond, which is between the Cys-345 residues of the two subunits [33,35]. However, although some HMW PAPs has a cysteine residue around position 120, it cannot form a linkage between two subunits [2,36]. Considering the broad range of substrates utilized by PAPs, nevertheless, it can be understood that the obviously different structure organizations among PAPs presumably reflected their diverse biochemical properties like substrate specificity and activities. Particularly, the “YERTC” located adjacent to the conserved metal binding motif “GHVH” in active site, suggesting that this conserved motif might be essential for the catalytic activity of PAPs.

PAPs with the phytase activity have been described [23,24,25,37]. Among the four groups, subgroup I-2 PAPs have been characterized as phytases, including AtPAP15, OsPAP23 (i.e., OsPHY1), GmPAP19 (i.e., GmPhy). Phytases are phosphatases, which can initiate the sequential hydrolysis of the phytate, a salt form of phytic acid (InsP6) that cannot be digested by humans and monogastric animals. Moreover, phytate can lead to the reduced availability of iron and zinc by forming strong complexes with the metals. Thus, it is considered as the most serious antinutritional compound for the absorption of a range of essential micronutrients including Zn^2+^, Ca^2+^, and Fe^2+^ [38]. Therefore, reducing the phytate content is significantly important for improving the micronutrient bioavailability, eventually benefiting human health. Unfortunately, tea products including different brand-pocketed roasted teas, tea leaves, green tea and green tea bags contain a significant amount of (more than 20 mg g^−1^) phytic acid. It is plausible that the CsPAP members in subgroup I-2 are phytases or enzymes with similar functions as phytases due to phylogenetic relatedness, which merits further characterization.

The redox properties of iron make it an essential element for virtually all organisms but is toxic when present in excess because Fe^2+^ catalyzes the generation of ROS. Tea plant is mainly cultivated on acidic soils, where bioavailable iron excess commonly occurs due to the biochemical characteristics of the element [29]. Transcriptome analysis results revealed that the transcript abundances of a large number of genes involved in the oxidation-reduction process and photosynthesis were changed by excess iron (Figure 3, Figures S2 and S3). A subgroup of 385 genes categorized to a significantly enriched GO term “oxidation-reduction processes” were upregulated such as peroxidase encoding genes. Peroxidases modulate ROS production by regulating H_2_O_2_ levels [39]. Our results indicated that many genes were induced to detoxify reactive oxygen species triggered by excess iron. An important sink tissue for iron is the leaves, where it is required for the synthesis of chlorophyll and the electron transport chains of the respiration and photosynthesis. Iron toxicity could inhibit photosynthesis by causing damage to the PS II reaction centre [40]. Our data illustrated that genes belonging to biological process categories “photosynthesis” and “carbohydrate metabolic process”, and genes located in the cellular component category “photosystem I”, “photosystem II” and “thylakoid”, were downregulated. This finding is in accordance with the repression of photosynthesis by excess Fe. An efficient regulatory system that adjusts plants to cope with excessive Fe is crucial for survival. The precise molecular mechanisms of plant adaptation to the high available iron are yet to be known. Transcription factors (TFs) are key regulators of iron homeostasis [21]. A previous study has shown that iron toxicity elicits strong ethylene signaling [41]. It is worth mentioning that many transcription factors were induced by excess iron, including WRKY, GATA and ethylene-responsive transcription factors (Figure S2, Supplementary Data set). The high Fe induced TFs might play important roles for efficient defenses of the plant against toxicity.

Studies have demonstrated that plant PAPs have a number of biological functions associated with carbon metabolism [12], cell wall synthesis [13], ROS scavenging [15] and phosphorus metabolism [42]. However, little information is available on whether PAPs, as iron-containing enzymes, are involved in plant iron homeostasis. We therefore explored the expression profiles of *CsPAP* genes both under normal and high iron supply conditions. Our study revealed that some *CsPAP*s constitutively expressed in roots and leaves (Figure 4), suggesting their general metabolic functions. Whereas some *PAP*s showed a biased expression profiling between roots and leaves, indicating their diverse organ-specific functions. The transcripts of two *PAP*s were not detected in either organ, which might imply that they are pseudogenes. Nevertheless, it cannot be ruled out at present that they could be expressed in other organs or be activated under a particular stimulus.

PAP has a tight link with iron because all known PAPs require Fe III- Fe II/Zn II/Mn II to form the enzymatic site. It has long been known that mammalian PAP functions as an iron carrier [10]. Few studies suggested that *PAP* genes might have important roles in iron homeostasis in the model plant *Arabidopsis*. The overexpression of *AtPAP15* increased foliar ascorbate [23]. Ascorbate chemically reduces iron(III) which is an obligatory step for the uptake of iron(II) in plants [43]. As iron(II) can promote ROS in excess conditions, plants must maintain a strict iron balance to cope with excess iron stress. It is tempting to assume that the repression of *CsPAP15a*, a homolog of *AtPAP15*, by high Fe might reduce the iron(II) concentration by decreasing the content of ascorbate. Furthermore, it was found that the iron content was significantly increased in the *AtPAP23* overexpression of *Arabidopsis* plants [22]. It could be speculated that the downregulation of *CsPAP23* might decrease the iron content, although its mechanism is not known. Moreover, *AtPAP10* was shown as significantly downregulated by iron deficiency [44]. In this study, *CsPAP10a* was shown as significantly upregulated, together with the iron storage protein gene *FER1*, by excess iron. It has been shown that the loss of ferritin proteins in leaves did not induce a significant modification of Fe content or any macroscopic phenotype in Fe excess conditions [45]. Ferritin-free chloroplasts were still able to accumulate Fe in conditions of excess and, more interestingly, that this loss of ferritins provoked an important modification of the iron distribution at the cellular level with a strong accumulation in the cell walls. Cell wall components, mostly hemicellulose and pectin, can bind iron. Thus, a large pool of iron is stored in cell walls, which contribute to the dynamics utilization of the iron [46]. Interestingly, PAP10 is a cell wall-bound protein. It is possible that PAP10 might function in distributing Fe in the cell wall to avoid its over accumulation in high iron conditions, whereas reducing the cell wall allocation in iron deficiency conditions. Hence, based on above clues, it is tempting to speculate that some *PAP*s are likely important players in iron homeostasis through the fine regulation of iron allocation, storage, and remobilization. The physiological and biochemical functions of Fe-responsive *PAP*s and their regulatory mechanisms are waiting to be fully uncovered.

## 4. Materials and Methods

### 4.1. Database Search of PAP Genes in the Tea Plant Genome

Identification of *PAP* genes was conducted by the keyword and BLASTP search. Initially, the “purple acid phosphatase” was used as the query to search the tea plant genome (http://www.plantkingdomgdb.com/tea_tree/) [47], and 21 genes were hit. In addition, protein sequences of the 29 *Arabidopsis* PAPs [5] were retrieved from The *Arabidopsis* Information Resource (TAIR) website (TAIR10, www.arabidopsis.org) and used to search the predicted protein sequences with BLASTP at the tea plant genome database website (http://www.plantkingdomgdb.com/tea_tree/) [47] and 53 predicted protein sequences were chosen based on the E value ≤ e^−10^. In total, 62 predicted PAP proteins were obtained by these two approaches. Next, the amino acid sequences of the 62 predicted PAP proteins were retrieved and the conserved sequence motifs (DXG/GDXXY/GNH(E/D)/VX2H/GHXH) were determined. As a result, 19 proteins containing the conserved sequence motifs were identified. Finally, in order to find all potential PAPs in the tea plant, each of these 19 PAPs was used as a query sequence for another round of BLASTP search at the same database. However, no more predicted PAPs were found. Taken together, our results suggested that there are in total 19 predicted PAPs in the tea plant genome, being named according to their homolog to *Arabidopsis* PAPs, with the prefix ‘CsPAP’ for *Camellia sinensis* PAPs.

### 4.2. Clustering and Bioinformatics Analysis of Tea Plant PAPs

The protein sequences of 19 CsPAPs were predicted from the tea plant genome database. To build a clustering scheme for plant PAPs, the protein sequences of PAPs from *Arabidopsis* [5], soybean [4], rice [7], maize [6], physic nut [8] and chick pea [3] and CsPAPs with at least three conserved motifs were aligned using the program Clustal Omega (https://www.ebi.ac.uk/Tools/msa/clustalo/) [48]. A phylogenetic tree was constructed using the neighbor-joining method with 1000 bootstrap replicates in the MEGA 7 program and the pairwise deletion was applied to deal with gaps or missing data in sequences [49]. The distance between sequences was estimated after the Poisson correction. The sequence logo of the motif was identified using the MEME suite 5.0.5 (http://meme-suite.org/tools/meme) [50].

The molecular weight and isoelectric point of the protein were predicted by the ProtParam (http://expasy.org./tools/protparam.html) [51]. Signal peptide was predicted using the SignalP4.1 server (http://www.cbs.dtu.dk/services/SignalP/) [52] using default settings. The subcelluar location predication of CsPAP proteins was conducted using the TargetP1.1 server (http://www.cbs.dtu.dk/services/TargetP/) using default parameters [53]. The N-glycosylation site of the protein was predicted by the NetNGlyc 1.0 server (http://www.cbs.dtu.dk/services/NetNGlyc/).

### 4.3. Plant Materials and Treatments

Two-year-old tea (*C. sinensis* var. Longjing 43) plants were transplanted into pots filled with inerts substrate perlite. Plants were fertilized with a half-strength nutrient solution in the first week and then grown for another week in a full-strength solution containing (in mM): (NH_4_)_2_SO_4_, 0.713; NH_4_NO_3_, 0.73; KH_2_PO_4_, 0.1; K_2_SO_4_, 0.46; CaCl_2_, 0.5; MgSO_4_, 0.41; Fe-EDTA, 0.032; H_3_BO_3_, 0.046; CuSO_4_, 0.002; MnSO_4_, 0.09; Na_2_MoO_4_, 0.0026; ZnSO_4_, 0.0091, pH was adjusted to 4.2 by 1 M H_2_SO_4_ [54]. For the expression analysis of *CsPAP*s in the different growth conditions of tea plants, the seedlings were then treated with different Fe concentrations (0.032 mM and 32 mM Fe-EDTA). Plants were cultivated in a growth chamber and were watered with deionized water every two days to maintain field capacity. At the 10th day after treatment, roots and young leaves were harvested separately. All tissue samples were stored at −80 °C for RNA extraction. Three biological replicates were conducted for each experimental condition.

### 4.4. RNA-seq Analysis of Tea Plants Grown Under Normal and High-Iron Conditions

Tissues were ground in liquid nitrogen and total RNA was extracted using the Pure Link RNA Mini Kit according to the manufacturer’s instructions (Ambion by Life Technologies, Carlsbad, CA, USA). The RNA purity was checked using the NanoPhotometer^®^ spectrophotometer (IMPLEN, Westlake Village, CA, USA). The RNA concentration was measured using a Qubit^®^ RNA Assay Kit in Qubit^®^2.0 Flurometer (Life Technologies, Carlabad, CA, USA). The RNA integrity was assessed using the RNA Nano 6000 Assay Kit of the Bioanalyzer 2100 system (Agilent Technologies, Palo Alto, CA, USA). The mRNA was purified from 3 µg of total RNA using poly-T oligo-attached magnetic beads and used to generate library for RNA sequencing using NEBNext^®^UltraTM RNA Library Prep Kit for Illumina^®^ (NEB, Ipswich, MA, USA) following the manufacturer’s recommendations. The library quality was assessed on the Agilent Bioanalyzer 2100 system. The clustering of the index-coded samples was performed on a cBot Cluster Generation System using TruSeq PE Cluster Kit v3-cBot-HS (Illumia, San Diego, CA, USA) according to the manufacturer’s instructions. After the cluster generation, the library preparations were sequenced on an Illumina HiSeq2500 platform and 125 bp/150 bp paired-end reads were generated.

Raw reads in the fastq format were first processed through in-house perl scripts. Clean reads were obtained by removing reads containing adapter, reads containing ploy-N and low quality reads from raw data. All the downstream analyses were based on the clean data with high quality. Reference genome and gene model annotation files were downloaded from the genome website directly (https://genome.jgi.doe.gov/portal/pages/dynamicOrganismDownload.jsf?organism=Phytozome#). Paired-end clean reads were aligned to the reference genome using Hisat2 (v2.0.5) [55]. The sequencing data were deposited to the Short Read Archive (SRA) database at the National Center for Biotechnology Information (NCBI) under the accession number PRJNA528567.

### 4.5. Quantification of Gene Expression Level and Alternative Splicing(AS) Analysis

Feature Counts v1.5.0-p3 was used to count the read numbers mapped to each gene [56]. Followed, FPKM (Fragments Per Kilobase of transcript sequence per Millions base pairs sequenced) of each gene was calculated. Differential expression analysis of two conditions (three biological replicates per condition) was performed using the DESeq2 R package (1.16.1) [57]. The resulting P-values were adjusted using the Benjamini and Hochberg’s approach for controlling the false discovery rate. Genes with an adjusted *p*-value < 0.05 and fold change > 2 were considered as differentially expressed. Further, the DEGs were used for gene ontology (GO) enrichment analyses according to the previous description [58]. GO terms were sorted based on the corrected *p*-value, and the corrected *p*-value < 0.05 was used as the significance cut-off. The rMATS (3.2.5) software was used to analyze the AS event [59].

## 5. Conclusions

We found two cysteine-containing motifs “GGECGV” and “YERTC” that specifically conserved in group II members, indicating that despite the presence of conserved sequence motifs, multiple genes in the plant *PAP* family can differ from each other in their overall structures. Further investigations are required to reveal the influence of these motifs on the biochemical functions of PAPs. In addition, we provide the evidence that some *PAP* genes are possibly involved in the response to excess Fe. It will be interesting to dissect the biological functions of Fe responsive *PAP*s in future studies.

## Figures and Tables

**Table 1:** Conserved residues and domains for identified putative CsPAPs.

Gene ID	Proposed Name	PAP Conserved Residues					Conserved Domain ^a^		
		GDXG	GDXXY	GNH(D/E)	VXXH	GHXH			
CSA004804	CsPAP2		GDISY	GNHE	VQGH	GHVH	MPP_PAPs		
CSA005153	CsPAP15b	GDLG	GDVTY	GNHD	ATWH	GHVH	MPP_PAPs	Pur_ac_phosph_N	
CSA006013	CsPAP1	GDLG	GDICY	GNHE	FLAH	GHVH	MPP_PAPs	Pur_ac_phosph_N	
CSA006536	CsPAP29a	ADMH	GDNIF	GNHD	VYFH	GHDH	MPP_Dcr2		
CSA006537	CsPAP29b		GDNIF	GNHD	VYFH	GHDH	MPP_Dcr2		
CSA006539	CsPAP29c	ADMH	GDNIF	GNHD	VYFH	GHDH	MPP_Dcr2		
CSA008696	CsPAP12	GDLG	GDLSY	GNHE	VLMH	GHVH	MPP_PAPs	Pur_ac_phosph_N	
CSA011989	CsPAP15a	GDLG	GDATY	GNHD	ASWH	GHVH	MPP_PAPs	Pur_ac_phosph_N	
CSA018804	CsPAP27a	GDMG	GDLSY	GNHE	FAAH	GHVH	MPP_PAPs	Pur_ac_phosph_N	Metallophos_C
CSA019400	CsPAP3b		GDNFY	GNHD	VVGH	GHDH	MPP_ACP5		
CSA021776	CsPAP3c	GDMG	GDISY	GNHE	FAGH	GHVH	MPP_PAPs	Pur_ac_phosph_N	
CSA026817	CsPAP27c	GDMG	GDIVY	GNHE	FIAH	GHVH	MPP_PAPs	Pur_ac_phosph_N	
CSA027593	CsPAP27b	GDMG	GDLPY	GNHE	FAAH	GHVH	MPP_PAPs	Pur_ac_phosph_N	
CSA028799	CsPAP10b	GDLG	GDLSY	GNHE	VLMH	GHVH	MPP_PAPs	Pur_ac_phosph_N	
CSA030189	CsPAP6a	GDLG	GDLSY	GNHE	VILH	GHVH	MPP_PAPs	Pur_ac_phosph_N	
CSA030190	CsPAP6b	GDLG	GDLSY	GNHE	VILH	GHVH	MPP_PAPs	Pur_ac_phosph_N	
CSA031017	CsPAP10a	GDLG	GDLSY	GNHE	VLLH	GHVH	MPP_PAPs	Pur_ac_phosph_N	
CSA034553	CsPAP3a	GDWG	GDNFY	GNHD	VVGH	GHDH	MPP_ACP5		
CSA036115	CsPAP23	GDLG	GDLTY	GNHE	AAWH	GHVH	MPP_PAPs	Pur_ac_phosph_N	Metallophos_C

Conserved residues are underlined. ^a^: MPP_PAPs, purple acid phosphatases of the metallophosphatase superfamily, metallophosphatase domain; MPP_Dcr2, Saccharomyces cerevisiae DCR2 phosphatase and related proteins, metallophosphatase domain; MPP_ACP5, Homo sapiens acid phosphatase 5 and related proteins, metallophosphatase domain; Pur_ac_phosph_N, Purple acid Phosphatase, N-terminal domain; Metallophos_C, Iron/zinc purple acid phosphatase-like protein C.

**Table 2:** General information for the 19 CsPAP genes and their deduced proteins.

Gene	Scaffold	Location (Start-Stop)	Length (aa)	Predicted Size (kDa)	Signal Peptide Length	Subcellular Location ^#^	Transmembrane Domain Site	Predicted Number of N-Glycosylation Site
CsPAP2	Sc0000741	607594-608772	392	44.0	No	_	350-371	2 *
CsPAP15b	Sc0002098	204497-207829	572	64.5	24	S	313-333	9
CsPAP1d	xpSc0055840	6841-16105	700	78.8	22	S	no	6
CsPAP29a	Sc0000663	233422-236333	387	42.1	29	S	no	1
CsPAP29b	Sc0000663	239793-242521	304	33.4	No	_	no	1 *
CsPAP29c	Sc0000663	250447-253464	387	42.2	29	S	no	2
CsPAP12	Sc0000340	248681-252365	469	53.8	24	S	no	4
CsPAP15a	Sc0000525	77490- 80649	398	45.5	No	_	no	4 *
CsPAP1a	Sc0001074	222761-234424	761	86.1	No	_	no	7 *
CsPAP3b	Sc0001483	375068-377024	267	30.5	No	_	no	0 *
CsPAP3c	Sc0000211	481949-485198	407	45.04	No	_	no	1 *
CsPAP1c	Sc0001926	105169-108133	522	59.1	No	M	no	2 *
CsPAP1b	Sc0002467	157423-166705	631	71.1	No	S	no	7 *
CsPAP10b	Sc0002450	126798-130541	364	41.8	21	S	no	4
CsPAP6a	xpSc0053256	290822-293152	402	46.5	22	_	no	2
CsPAP6b	xpSc0053256	310322-313230	472	54.6	22	S	no	4
CsPAP10a	xpSc0053660	137781-142512	777	89.0	No	_	no	5 *
CsPAP3a	Sc0004847	59846-70900	370	42.0	No	C	no	1 *
CsPAP23	Sc0001836	97264-106048	683	76.0	23	S	656-677	5

^#^: C, Chloroplast; M, Mitochondrion; S, Secretory pathway; _, Any other location; * Proteins without signal peptides are unlikely to be exposed to the N-glycosylation machinery and thus may not be glycosylated (in vivo) even though they contain potential motifs.

## Supplementary Materials

Supplementary materials can be found at https://www.mdpi.com/1422-0067/20/8/1954/s1.
