# Supplementary material for: Identification of Tea Plant Purple Acid Phosphatase Genes and Their Expression Responses to Excess Iron
Source: Int J Mol Sci. 2019 Apr 21;20(8):1954. doi: 10.3390/ijms20081954 (PMC6515233; doi:10.3390/ijms20081954)
Supplement: Supplementary file 1 [file ijms-20-01954-s001.zip › Table S3.pdf]

Supplementary Table 3. CsFER1 cDNA sequence

5'-CTAATACGACTCACTATAGGGCAAGCAGTGGTATCAACGCAGAGTACATGGGGATACCTTGA
CACCTCCATTGATCATTTTAGCTAAGTCTGCCCTTAATTCAAACCTCCATATTCTTGTAAATCTC
TCTCTCTCTCTCTCTCTCTCTCTTCTCTAAAATCATGTTTCCGCAGGCAACTTGTCCGGCTGTTT
CTCTCTCTTCCATGCACGGGGTTAGTCTTAGACTTGGACTACCCTTATCATCACATCCTAGCTTC
AGCTCACCTTCCTCATCATCTGTTACGAGTTTTGGGGACAAAAAGAGGGGTTATGGGTTCGTTGT
CTCGGCTTCAACGGAGACTAGTTTGGCGCAGACAGGTGTGATCTTCAAACCATTTGAAGAGGTCA
AGAAAGAAGATTTCATGATCCCTATCTCCCGCCAGATTTCGCTTGCTCGCCAGAGGTACCAAGAT
GAGTGCGAAGCTGCGATCAACGAACAGATCAATGTGGAATACAATGTGTCGTATGTTTATCACGC
CATGTACGCATACTTCGACAGGGACAATGTCGCTCTCAAGGGCCTTGCAAAATTTTTCAAGGAGT
CAAGTGATGAAGAAAGAGAACATGCTGAGAAATTTATGAAATACCAGAACATACGCGGAGGAAGA
GTTAAGCTACACTCTATACTGGCGACCCTTTCTGAATTTGAACATGCTGAGAAGGGTGATGCTTT
GTATGCAATGGAACTAGCACTGTCCTTGGAGAAGTTGACAAATGAGAAACTTCTGAGCTTGCATG
GTGTGGCTGCTCAAAACAATGATCCTCAAATGACTGAGTTTATTGAGCGTGAATTTCTAGCTGAG
CAGGTGGAAGCAATTAAGAAAATTGCAGACTTTGTCACTCAGTTGAGACTGGTTGGGAAAGGACA
TGGGGTGTGGCACTTTGATCAGAAGCTCCTACACGAGGGTGATGGTGTTTTTTGAAGATTTCCAT
CCATGTTTTCTTTTTCGGTTAGAGTCTATGTTCCATAAAACCTACGGTGTGATGATTTACAAACT
TTTGTGTTAATATTCAGATGAATGAACAAATCAACTCAAACCCAAAAGAAGTAAACTTTGTTATT
GTGGAAAACACAATAAATGTAATATAAAGTGGGTGTTTGAGAAAAAAAAAAGTAAAAAAAAAAAA
AAAAAAAAAAAAAGTACTCTGCGTTGATACCACTGCTT-3'
