# Supplementary material for: Identification of Tea Plant Purple Acid Phosphatase Genes and Their Expression Responses to Excess Iron
Source: Int J Mol Sci. 2019 Apr 21;20(8):1954. doi: 10.3390/ijms20081954 (PMC6515233; doi:10.3390/ijms20081954)
Supplement: Supplementary file 1 [file ijms-20-01954-s001.zip › ijms-489502 supplementary figures.pdf]

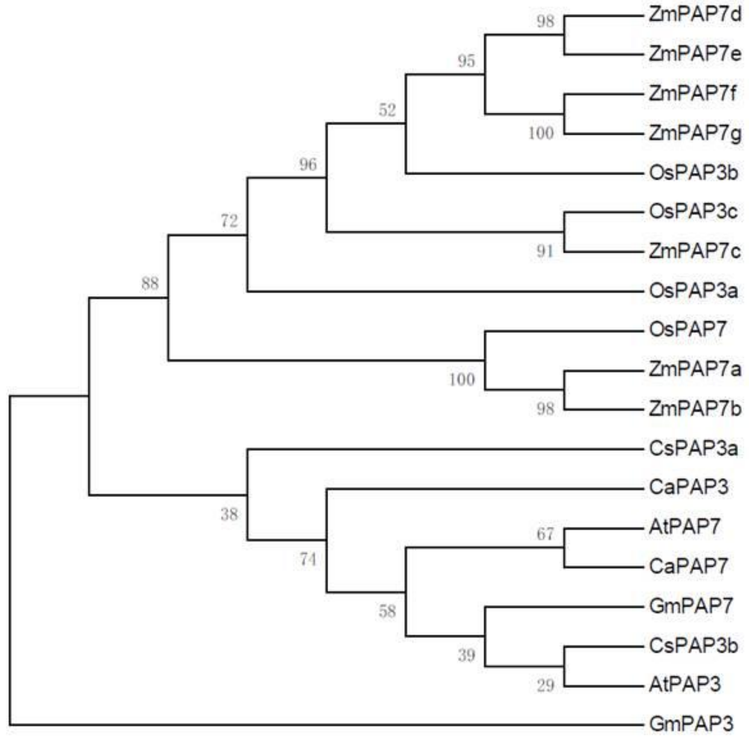

Figure S1. Phylogenetic relationships of PAP3 and PAP7 to homologous proteins from monocots and dicots in groups.

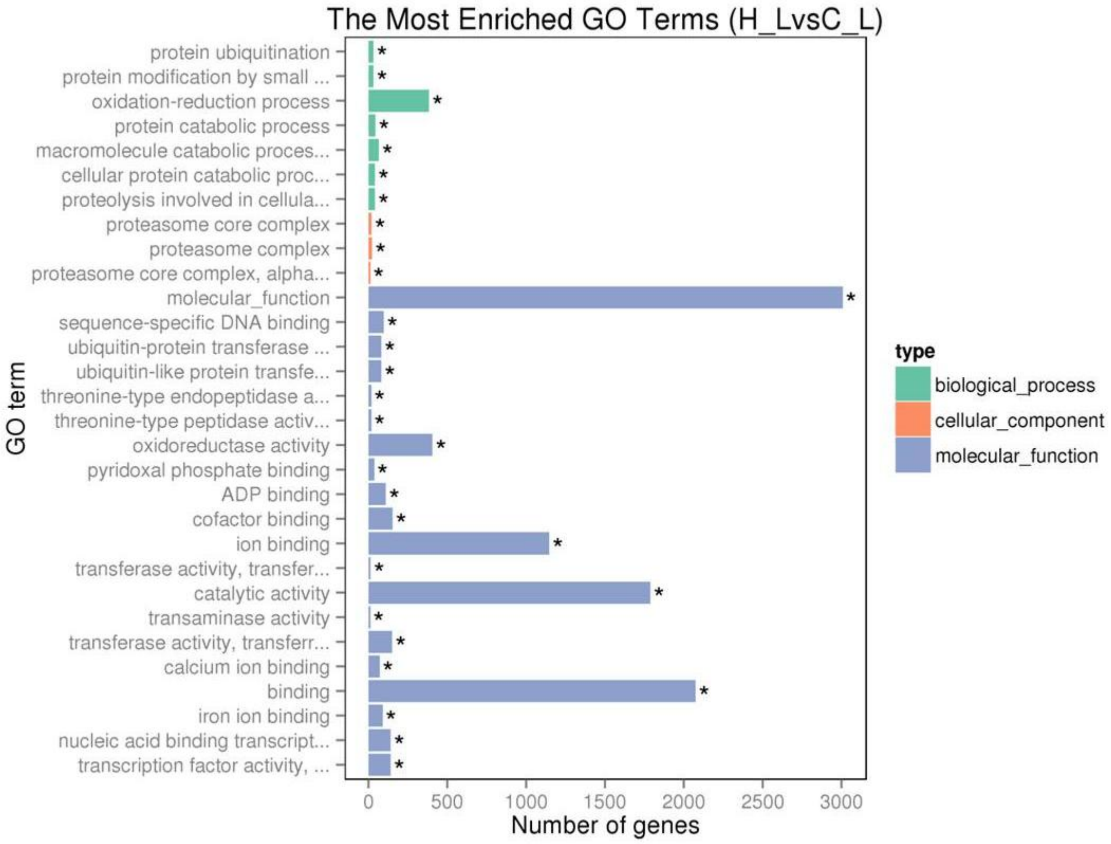

Figure S2. GO enrichment analyses of the upregulated DEGs by iron excess. * GO terms that were significantly enriched in the upregulated DEGs were summarized in three main categories: Biological process (green), molecular function (blue), and cellular component (orange). The x-axis indicates gene numbers in each term.

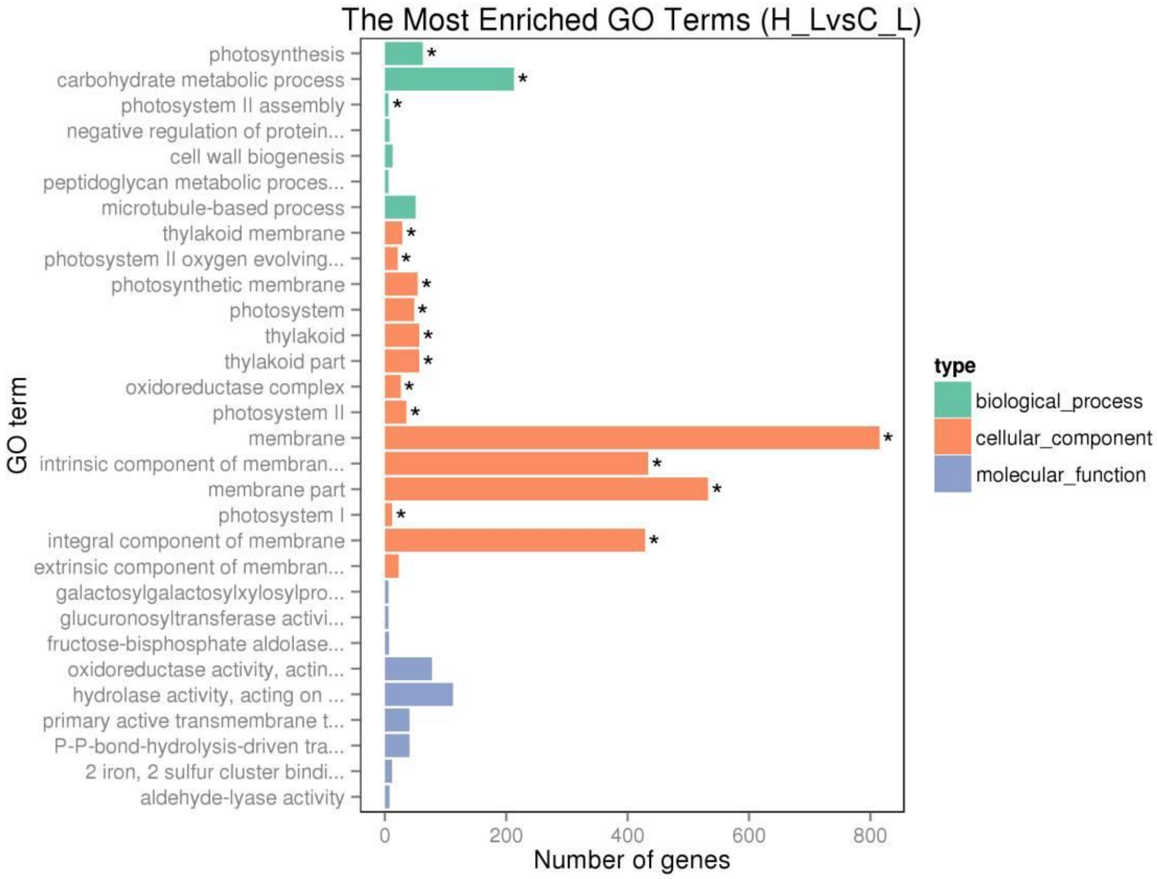

Figure S3. GO enrichment analyses of the downregulated DEGs by iron excess. * GO terms that were significantly enriched in the downregulated DEGs were summarized in three main categories: Biological process (green), molecular function (blue), and cellular component (orange). The x-axis indicates gene numbers in each term.

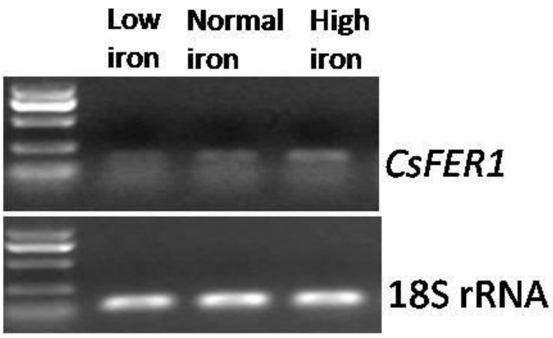

Figure S4. The transcript abundance of CsFER1was increased by excess iron supply.
