# Supplementary material for: Identification of Tea Plant Purple Acid Phosphatase Genes and Their Expression Responses to Excess Iron
Source: Int J Mol Sci. 2019 Apr 21;20(8):1954. doi: 10.3390/ijms20081954 (PMC6515233; doi:10.3390/ijms20081954)
Supplement: Supplementary file 1 [file ijms-20-01954-s001.zip › Table S1.pdf]

Supplementary Table 2. Sequences of identified CsPAPs.

>CSA004804.1 CSA004804 Sc0000741:607594-608772(+)
MGAATPYSTFLRTQDESISTMKWISRDIEALGDKPSFVSHIGDISYARGYSWLWDTFFAQ
IEPVASEVPYHVCIGNHEYDWPHQPWKPDWSYSVYGKDGGGECGVPYSLRFNMPGNSSEV
TGTRAPATRNLYYSFDFGSVHFVYISTETNFLPGSSQYNFIKHDLESVDRKKTPFVVVQG
HRPMYTTSNENRDAPFRERLQEHLEPLFVKNKVTLALWGHVHRYERFCPMNNFTCGNMDL
NGEDSEGFPVHIVIGMAGQDWQPIWEPRADHPNDPIFPQPGRSLYRGGEFGYTRLVATKE
KLTFSYVGNHDGEVHDTVEILASGHVLNGGGSGDDGSTASSTMMESTFSWYVKGASVLVL
GAFVGYVIGYISHARRGAASGNNWTPVKNEEI*

>CSA005153.1 CSA005153 Sc0002098:204581-207829(+)
MGFHVVTVIGLPLFLLLSLVLAHAEIPTTLEGPFKPVTVPLDNNFRGLATDLPDTDPRVQ
RKVTGFEPEQISVSLSSNHDSVWISWITGEFQIGNNIEPLNPETVRSVVYYRKSKSALTH
KAKGDSLIYNQLYPFEGLQNYTSGIIHHVRLTGLKPNTVYYYQCGDPSLPAMSNVSYFKT
MPVSGPRNYPKRIAVVGDLGLTYNTTSTISHLMRNRPDLVLLVGDVTYANLYLTNGTGSD
CYSCSFPQTPIHETYQPRWDYWARFMQPLVSKVPIMVIEGNHDIEQQAGNLTFASYSSRF
AFPSEESESSSTLYYSFNAGGIHFIMLGGYIAYNKSADQYKWLERDLAKVDRKVTPWLVA
TWHPPWYSSYKAHYREAECMKVAMEELLYKYGVDMVFNGHVHAYERSNRVYNYTLDPCGP
VYITVGDGGNREKMAIPHADEPGNCPDPSTTPDGYMGGFCAYNFTSGPAAGKFCWDRQPD
YSAYRETSFGHGILEVKNETHALWSWHRNQGMYNMAGDQIYIVRQPDRCPVGPKCSYWSG
NTTSAGSIHLMTSALIGLGTRRQRTKFVLWDD*

>CSA006013.1 CSA006013 xpSc0055840:7657-15883(+)
MTLSGLILLAILSGFNGATSHGDQQPLSRIAIQRAVFAFDDHAYIKASPPVLGLEGQNSE
WLTLEYSIPNPSIDDWIGVFSPANFSASTCPPENGRVFPPLLCTAPIKVRAYQYANYTNP
EFKDNGKGSLRLQLIKQRSDFSFALFSGGLLTPKLVAVSNIVDFANPNAPVYPRLAQGKT
WNEMTVTWTSGYGINEAEPFVEWGPKGGEQSRSPAGTVTFDRRSAPARTVGWRDPGFIHT
SFLKDLWPNSVYTYKIGHMLLNGTYIWTRMFQFRASPYPGQNSVQRVVIYGDLGKDEVDG
SSEYNNFQRGSLNTTKQLIKDLKNTDIVFHIGDICYANGYLSQWDQFTSQIEPIASTVPY
MIASGNHERDWPGTGSFYQNMDSGGECGVLAETMFYVPTENRAKFWDMYEGVVTTIRSPA
GETNEFPITGRLSQQQGRLMRMWPIGLRRDGVSGEVRLGYCAISEYPLDLKDRMKKKDSC
SLPHLIGLKAWPGLVWYSTDYGMFKFCIADTEQDWREGSEQYVFIEHCLASVDRLKQPWL
IFLAHRVLGYSSDANYALEGSFAEPMGRDSLQKLWQKYKVDIAMFGHVHNYERTCPIYQN
ICTNKEKHYYKGPLNGTIHVVAGGAGASLSPFTTIQTHWSIFRDFDYGFVKLTAFDHSNL
LFEYRKSRDGNVYDSFRISRDYRDILACTVDSCPSSTLAS*

>CSA006536.1 CSA006536 Sc0000663:233422-236241(-)
MANVHDAVVRLVVALLVVTLLSGPICIFGKQSQSALRFDRNGEFRILQVADMHYGDGKTT
PCEDVLPGQVRGCSDLNTTAFLKRMILAFNPHLIVFTGDNIFASDATDAASSMNAGFAPA
VSANIPWVAVLGNHDQESTLSREGVKKHIVGMKNTLSQLNPPEVHVIDGFGNYNLEIHGI

EGSSFANKSVLNLYFLDSGDYSTVPSIPGYGWIKPSQQFWFQRTSMKLRRAYMNKPEAQK
APAPGLVYFHIPLPEYASFDSSNFTGVKQESISSPSVNSGFFTTMVEAGDVKAVFTGHDH
VNDFCGELTGIHLCYAGGFGYHAYGKAGWARRARVVVASMEKTEKGGWGAVKSIKTWKHL
DDEKLTTIEGQVLWSKSSVGNASSLVP*

>CSA006537.1 CSA006537 Sc0000663:239793-242521(-)
MCCKYLLGWNAGDNIFGFDATDAASSMNTAFAPAVLANIPWVAILGNHDQESTLSREGVM
KHIVGLKNTLSQLNPPEVHVIDGFGNYNLEIHGIEGSTFANKSVLNLYFLDSGDYSTVPS
IPGYGWIKPSQQFWFQRTSMKLRRAYMNKPEAQKAPAPGLVYFHIPLPEYASFDSSNFTG
VKQENISSPSVNSGFFTTMVEAGDVKAVFTGHDHVNDFCGELTGIHLCYAGGFGYHAYGK
AGWARRARVVVASMEKTEKGGWGAVKSIKTWKHLDDEKLTTIEGHVLWSKSSVGDRRKKQ
IGRA*

>CSA006539.1 CSA006539 Sc0000663:250558-253377(-)
MANVHEAVVRLVVALLVVTLLSGPICIFGKQSQSALRFDRNGEFRILQVADMHYGDGKTT
PCEDVLPGQVRGCSDLNTTAFLKRMILAFNPHLIVFTGDNIFASDATDAASSMNAGFAPA
VSANIPWVAVLGNHDQESTLSREGVKKHIVGMKNTLSQLNPPEVHVIDGFGNYNLEIHGI
EGSSFANKSVLNLYFLDSGDYSTVPSIPRYGWIKPSQQLWFQRTSMKLRRAYMSKPEAQK
APAPGLVYFHIPLPEYSSFDSSNFTGVKQENISSPSVNSGFFTTMVEAGDVKAVFTGHDH
VNDFCGELTGIHLCYAGGFGYHAYGKAGWARRARVVVASMEKTEKGGWGTVKSIKTWKHL
DDEKLTAIEGQVLWSKSSVGNASSLVP*

>CSA008696.1 CSA008696 Sc0000340:249354-252365(-)
MVPKWVCAGFVYLLLGLILNVAEFCNGGMTSGYQRPNNLSFDMPLDSDVFRVPPGYNAPQ
QVHITQGDYEGRGVIISWITPDEPGSNTVVYWAENTELKNCADGIVVTYKYFDYTSGFIH
HCTIKDLEFDTKYYYEVGIGNTTRQFWFITPPKVGPDVPYTFGLIGDLGQTYDSNTTLTH
YELNPAKGQALLFVGDLSYADDYPFHDNNRWDTWGRFIERNAAHQPWIWTAGNHEIDFAP
AIGESIPFKPYTHRFFVPYEASESTSPLWYSIKRASAYIIVMSSYSAFGKYTPQYKWFTN
KVTEVNRTETPWLIVLMHPPMYNSYVSHYMEGETMRVMYEPWFVEYKVDVVFAGHVHAYE
RSERVSNVAYNIINGLCTPVNNHSAPVYITIGDGGNQEGLSTAMTQPQPHYSAYREASFG
HGIFDIKNRTHAYFSWHRNQDGYAIEADSQWFHNRYWKSSEESSVASSR*

>CSA011989.1 CSA011989 Sc0000525:77490-80649(+)
MLLPLSCLDKGLQPNTIYYYRCGDPSMGAMSEVFSFKTMPIHGPKIYPKRIAVVGDLGLT
YNTTSTVEHMIQNDPNLILMTGDATYADLYLTNGTGADCYFCAFGHTPIHETYQPRWDYW
GRYMQRVVAKVPIMMIEGNHDVEEQGGDVQFASYSSRFAYPSKESGSKSTFILRFPSFFV
AAVQYKWLKKDLAKVDREVTPWLVASWHAPWYNSYKAHYREAECMMIEMEELLYKNGVDL
VFNGHVHAYERSNRVYNYTLDPCGPVYITIGDGGNREDRAIPFADEPGSCPKPSSRLGNC
ATNFTSGPAAGKFCWDRQPDFSAYRESSFGHGILEVKNETHALWTWHRNQDMYNEIGDQI

YIVRQPDKCLVKPKPTKELGCWSQVTWEDVKREGFKHG*

>CSA018804.1 CSA018804 Sc0001074:222761-234424(+)
RLKELSYYDSSSTCPPDNSRQQAPYICSAPIKYKYANESSSDYTKTGNASLKFILINQRA
DFSFGLFTGGLSNMTVTWTSGYNIDEAVPFVEWGFKGEHQTQSPAGTLTFHRNSMCGSPA
RTVGWRDPGFIHTSFLKELWPNSVYTYKLGHRLFNGSYIWSKVYSFKSSPYPGQDSLQRI
IVFGDMGKAERDGSNEYSNYQPGSLNTTDQLIKDLDNFDIVFHIGDLPYANGYISQWDQF
TSMVEPIASTVPYMIASGNHERDTPGTGSFYDTNDSGGECGVLAETMFYVPAENRAKFWV
WDSKSVILDVRSSRVGLVMDVWSVDGVAIVGVKMTVTWTSGYNINEAVPFVEWGFKGEHQ
TQSPAGTLTFHRNSMCGSPARTVGWRDPGFIHTSFLKELWPNSVYTYKLGHRLFNYIWSK
VYSFKSSPYPGQDSLQHVVVFGDMGKAERDGSNEYNNYQPGSLNTTDQLIKDLDNIDIVF
HIGDLSYAKGYISQWDQFTSMVEPIASTVPYMIASGNHERDTPGTGSFYDTNDSGGECGV
LAETMFYVPAENRAKFWYSTDYGMFHFCIADTEHDWREGTEQYKFIEHCLASADRQKQPW
LIFAAHRVLGYSSNKYYALEGSFEEPMGRESLQKLWQKYKVDIAFFGHVHNYERICPIYQ
NQCVNTEKSHYSGTVNGTIHVVVGGGGSHLSEFSSLQTRWSLYRDYDFGFVKLTAFNHSS
LLFEYKKSSDGKVYDSFTISRDYRDVLACVHDGCEPTTLAT*

>CSA019400.1 CSA019400 Sc0001483:375243-377024(-)
MGIIGEKLDIDFVISTGDNFYEDGLMGINDPAFYDSFSNIYTAPSLQKTWYSVLGNHDYR
GDVEAQMSPILREKDKRWVCMRSFILNADIVEFFFVDTTPFVDKYFTDPGDNVYDWKGVL
PRKNYLSNLLNDVDLALKSSSAIWKIVVGHHTIKSAGQHGITEELVVNLLPILQENNVHV
YMNGHDHCLEHISCSKSKIEFLTSGGGSKAWRGNVKEWNPEEMKFYYDGQGFMSVQLDEA
KANFIYYDVFGEVLHKFMVSKEFHSTS*

>CSA021776.1 CSA021776 Sc0000211:481949-485198(+)
MRLTWVSGDKEPQQVQYGGGKSQTSQVTTFSPDNMCSSSILPSPAKDFGWHDPGYIHSAV
MTGLKPSSTFSYRYGSNSVGWSDQIQFTTPAVGGSDELRFLAFGDMGKAPRDASVEHYIQ
PGSLSVTKAMASEVASGNVDSIFHIGDISYATGFLVEWDFFLHLISPVASQVSYMTAIGN
HERDYVDSGSVYTTPDSGGECGVPYETYFPMPTPAKDKPWYSIEQGPVHFTVISTEHDWS
QNSEQYDWMNKDMASVDRSKTPWIIFAGHRPMYSSSSGILPSVDNNFVQAVEPLLLANKV
DLVLFGHVHNYERTCAVYQNECKAMPKKDGHGIDTYDNTNYSAPVQVVIGMAGFTLDKSP
ASADSWSLTRISEFGYVRVHATRQDLTFKYVNANTRKVEDSFRITKS*

>CSA026817.1 CSA026817 Sc0001926:105169-108133(-)
MLFLQYKYATAASSGYTKTGKTIIHFQLINQRSDFSFALFSGGLSNPKLVAISNPITFAN
PKAPLYPRIAQGKSWDVMTVTWTSGYNIDEAVPLVEWGFKGGNQIRSPAGTLRIHQSSMC

GGPARTVGWRDMGFIHTSYLKDLWPNTMYTYKMGHQLANGSIDWSKMYSFKSSPFIGQDS
LQRVIIFGDMGKVVPNPNKEQKGKEKQENGERDGSNEYSNYQPGSLNTTDQLIKDLNNYD
MVLHIGDIVYSNGYTSQWDQFTSQIEPIASTKPYMIASGNHERDWPGTGSFYDTMDSGGE
CGVLAETMFYVPAENRAKYSTDYGMFHFCIADSEHDWREGTEQYKFIEHCLASADRKKQP
WLIFIAHRVLGYSSDKFYGLEGSFEEPMGRESLQKLWQKYKVDMAFYGHVHNYERSCPVY
QSRCVTSDTSHFSGPVQGTIHVVVGGGGSHLSQFSELNTHWSLYKDYDWGFVKLTAFNHS
ALLFEYKKSRDGKVYDSFTISRDYKDVLACVHDSCPHVTLAT*

>CSA027593.1 CSA027593 Sc0002467:157423-166705(-)
MGHFPSQRFILTIFMSCSFFFFFFFYCVNSTSIFSATHLRGGSGEQPLSKIAIHNAVFAI
HSSASITASPFLLGKHGEDSQSITVKLKCPNPSEDDWVGVFSPANFNSSTCPPDNSRQQA
PYICSAPIKYKYANESSSDYTKTGNASLKFILINQRADFSFGLFTGGLSNPKLVAVSNVI
TFSNPKAPLYPRLAQGKSWNEMTVTWTSGYNIDEAVPFVEWGFKGEHQTQSPAGTLTFHR
NSMCGSPARTVGWRDPGFIHTSFLKELWPNSVYTYKLGHRLFNGSYIWSKVYSFKSSPYP
GQDSLQRIIVFGDMGKAERDGSNEYSNYQPGSLNTTDQLIKDLDNFDIVFHIGDLPYANG
YISQWDQFTSMVEPIASTVPYMIASGNHERDTPGTGSFYDTNDSGGECGVLAETMFYVPA
ENRAKFWYSTDYGMFHFCIADTEHDWREGTEQYKFIEHCLASADRQKQPWLIFAAHRVLG
YSSNKYYALEGSFEEPMGRESLQKLWQKYKVDIAFYGHVHNYERICPIYQNQCVNTEKSH
YSGTVNGTIHVVVGGGGSHLSEFSSLQTPWSLYRDYDFGFVKLTAFNHSSLLFEYKKSSD
GKVYDSFTISRDYRDVLACVHDGCEPTTLAT*

>CSA028799.1 CSA028799 Sc0002450:127167-130541(+)
MGLVRFFLGSALGVLGLFVNAVVLCNGGTTSAFVRQVEKTVDMPLESDVFHVPPGHNAPQ
QVHITQGDHEGKGLIVSWVTMDDPGSSTVLYWSENSKHKNKAKGTVVTYKFYNYTSGYIH
HCTIKNLEFDTKYYYEVGIGHTTRTFWFTTPPEVGPDVPYTFGLIGDLGQSYDSNMTLTH
YALNPTKGKTMLFMGDLSYADNYPNHDNVRWDTWGRFTERSAAYQPWIWTAGNHEIDFAP
EIGETKPFKPYTHRYRVPYKASNSTAPFWYSIKRASAYIIVLSSYSAYGKYTPQYKWLEA
ELPKVSRSETPWLIVLMHAPWYNSYNYHYMEGETMRVMYEPWFVKYKVDVVFAGHVHAYE
RSAL*

>CSA030189.1 CSA030189 xpSc0053256:290822-293152(-)
MATMWFWLMTCVTSTFVRKEWSTDMPESSFPPPPGTNAPEQVHITQGDHEGRSMIISWVT
PVEKYPNVVTYWEAGTKDLHKLQTHSEITTYRYYNYASGYIHHATIKDLKFDTKYFYEVA
SNEVTRQFSFTTPPKPGPDVPYTFGVIGDLGQTTDSXPYTFGVIGDLGQTEDSNRTLEHY
MSNPIKGQAVLFAGDLSYADDHPNHDNTKWDTFGRFIEKSVAYQPWIWSAGNHEIDLASN
LEETDPFKPYLHRYYVPYKASQSTSPLWYSIKRASTYIIVLSSYSAYGKYTPQYKWLEQE
LPKINRYETPWVIVILHAPWYNSNHYHYMEGESMRVQFEPWFVQYKVDIVFAGHVHSYER
SERISNIKYNITNGLSLPVRDINAPVYITIGDGGNIEGIANK*

>CSA030190.1 CSA030190	xpSc0053256:310322-313230(-)
MAIIWFWLMTCVLLHIAVLCNAGVTSGFVRKAAWSVDIPKSHFPPPSGTNAPEQVHITQG
DHEGRGMIISWVTPVDRYPNVVTYWEADTEHEHNKHKHYHKHKTHSTITTYRYYDYSSGY
IHHATIKHLKFDTKYFYEVASHNVTRRFSFTTPPKPGPDVPYTFGVIGDLGQTADSNQTL
EHYVSNPIKGQAVLFAGDLSYADDHPNHDNTKWDTFGRFIEKSVAYQPWIWSAGNHEIDF
APNLGESVPFKPYLHRYHVPYKASQSTSPLWYSIKRASTYIIVLSSYSAYGKYTPQYKWL
EQELPKINRSETPWIIVILHAPWYNSNHYHYMEGESMRVQFEPWFVQHKVDIVFAGHVHS
YERSERISNVKYNITNGLSGPVRDSSAPVYITIGDGGNIEGIADNFIDQQPSYSAYREAS
FGHAILEIKNRTHAYYTWHRNQDNEPVIADSAWFFNRFWYPEDESSINNKEA*

>CSA031017.1 CSA031017	xpSc0053660:137781-142512(-)
MTIVLSWEGLNPQPAHSIQMASMRSEADDWRRIRSIVKGLVSDSPLTYPSMDLLRLLFWN
CRGASNNNFKSNLTEIIRTHKPEILVLLETKVPFSKIGNFFNRLGSTASTIVDLGGRDTS
QVTVRASSATSQAIHATIHKADYDEWVLAAVYANPSPVMRDQLWDNLEDVAGTMGKPWLV
AGDFNDFATQGERKSFTSRNSTIRNQKFLDRVNNCNLMDLGSPGPSMTWTNNQKGLANTM
ERLDRAMCNSEWRTMFPEATVRILPRTYSDHSPLLVYTQGMHQLNPLNRPFRFEATWMTH
PGLIDVINSSWTSMNHNLIDSTTVFTCKVKEWNRELVLGFVWLNNWIKLNLIWVLSQREE
SLVCLDLGFGEVHITQGDHVGKAVIVSWVTMEEAGSSTVLYWSENSKNKNKAKGTVGRYK
FYSYTSGHIHHCTIKNLEFDTKYYYEVGIGHTARTFWFTTPPEVGPDVPYTFGLIGDLGQ
SYDSNKTLTHYESNPTKGKTVLFVGDLSYADTYPNHDNERWDTWGRFVERSTAYQPWIWT
AGNHEIDFAPDLGETKPFKPFTHRYHVPYKASNSVAPFWYSIKRASAYIIVLSSYSAYGK
YTPQYQWLLAELLKVNRSETPWLIVLLHAPWYNSYNYHYMEGETMRVMFEPWFVQHKVDV
VFAGHVHAYERSERVSNIAYNVVNGICSPVKDQSAPVYITIGDGGNLEGLATNMTEPQPK
YSAFREASFGHAIFDIKNRTHAYYSWHRNQDGNAVEADSLWFFNRIWHQVDDSTSTH*

>CSA034553.1 CSA034553	Sc0004847:59920-70900(-)
MILIVQLVHPGQRPGPPKARIPLGSGPGSGLNFLTRGPGLPMAGPTHLEFWREPERSDIG
HPSPGPELQRLEHHGKSDESLSFLVIGDWGRGGLYNQSEIAFQMGRIGEKLDIDFVISTG
DNFYDNGLKGVNDPAFQESFTKIYSQKSLQKPWYSILGNHDYRGDAKAQLNPMLRKIDSR
WICLRSFTLNTGSADFFFMDTTPFVDVYFTDPEERVYDWRGIYPREIYLNNLLKDLDSAL
RNSTAEWKIVVGHHAIRSISHHGDTLELIDQLLPVLEMNGVDFYVNGHDHCLQRISCPDN
RIQFLTSGAGSKAWRGDDKKHDKCAVHFFYDGQGFMSMQLKSTEAKMVFYDVFGQPIHQW
SVSRMLHSEM*

>CSA036115.1 CSA036115	Sc0001836:97465-106048(-)
MEFCKMGIASIVFFIFLDMVVIAQTIPTTLQGPFTPVTHSMDPSLRLGSDDLPMDNPRLK
RNVTAMFPEQIALALSSSSSMWVSWVTGTGLNWDSQIGLNVTALDPSTVASEVWYGKETG
DYSMKRNGVSTVYSQLYPYEGLWNYTSGIIHHVKIDGLEPETKYYYKCGDSSLPAMSDEQ
VFETLPLPGPNRYPRRIAVIGDLGLTSNSTTTIDHLIENDPSIVLMVGDLTYANQYQTTG
GKGASCFSCAFPNAPIRETYQPRWDAWGRFMEPLTSRVPMMVIEGNHEIETQAAGISFQS

YLTRFAVPSNESGSNSNFYYSFDAGGAHFIMLGAYVDYNSTGTQYAWLKEDLSKIDRSVT
PWLVAAWHSPWYNSYSSHYQEFECMRQQMEALLYQYGVDIVLSGHVHAYERMNRVYNYML
DPCGPVYITVGDGGNIEKVDVDHADDPGKCPSAGDNMPEFGGVCHLNFSSGPAKGNFCWD
KQPEWSAYRESSFGHGILEVHAYERMNRVYNYMLDPCGPVYITVGDGGNTEKVDVDHADD
PGKCPSAGDNVPEFGGVCHLNFSSGPAKGNFCWDKQPEWSAYRESSFGHGILEVVNSTYA
LWTWHRNQDIYKENSSGDQIYIVRQPQSCSVPSKNSEITSSPPMDSAAPSPPAMDAVAGF
GVHLSGAFITYFLFLIFMPLHWL*
